# 1859. Median Income by Location of Residence and Clinical Outcomes of Hospitalized Persons with COVID-19 in an Urban Veterans Affairs Medical Center

**DOI:** 10.1093/ofid/ofad500.1687

**Published:** 2023-11-27

**Authors:** Matthew Tuck, Heather Rivasplata, Steven Towers, Angelike P Liappis, Cherinne Arundel, Anca Dinescu, Zachariah Hamidi, Haitham Alaithan, Surabhi Uppal, Pratish C Patel, Shikha Khosla, Samuel Simmens, Debra A Benator

**Affiliations:** DC VAMC/ GWU, Washington, District of Columbia; Uniformed Services Univ of Health Sciences, Washington, District of Columbia; GWU Milken School of Public Health, WAshington, District of Columbia; Washington DC Veterans Affairs Medical Center , Washington, DC; DC VAMC / GWU, Washington, District of Columbia; DC VAMC, Washington, District of Columbia; BROOKE ARMY MEDICAL CENTER, WASHINGTON, District of Columbia; GWU, WASHINGTON, District of Columbia; Medstar Shah Medical Group, Oxon Hill, MD, washington, District of Columbia; Vanderbilt University Medical Center, Nashville, Tennessee; DC VAMC, Washington, District of Columbia; GWU Milken Institute School of Public Health, Washington, District of Columbia; Washington DC VA Medical Center, Washington DC, DC

## Abstract

**Background:**

The COVID-19 pandemic has disproportionately affected racial and ethnic minorities and those of low socioeconomic status, including higher rates of illness severity and death. This has been attributed in part to decreased healthcare access. We sought to determine whether place of residence as a marker of socioeconomic status, healthcare access, and median income were predictive of disease severity in a large integrated healthcare system.

**Methods:**

We conducted a prospective observational cohort study of ­348 patients admitted to the hospital with a diagnosis of COVID-19 between March 1, 2020 through June 30, 2021. Using univariate and multivariable logistic regression, we evaluated associations of disease severity outcomes with demographic characteristics of veterans, age-adjusted Charlson Comorbidity Index (CCI), and median income by place of residence.

**Results:**

< The mean age of our cohort was 68 and 90% were male. The racial and ethnic makeup was 83% black, 14% white, 5% Hispanic or Latino, and 3% other. In unadjusted models, only median income and the CCI predicted need for high-flow oxygen (p = 0.009 and p < 0.0001, respectfully) and death (p = 0.04 and p = 0.0005, respectively), while the CCI also predicted the need for intubation (p = 0.02). In multivariable models, CCI was strongly associated with the need for high-flow oxygen, intubation, and death (p = 0.0001, 0.01, and 0.02, respectively); age alone remained the most powerful predictor of death. Race and ethnicity were not associated with the need for high-flow oxygen, intubation, or death. Place of residence was not associated with worse outcomes.

**Figure:**
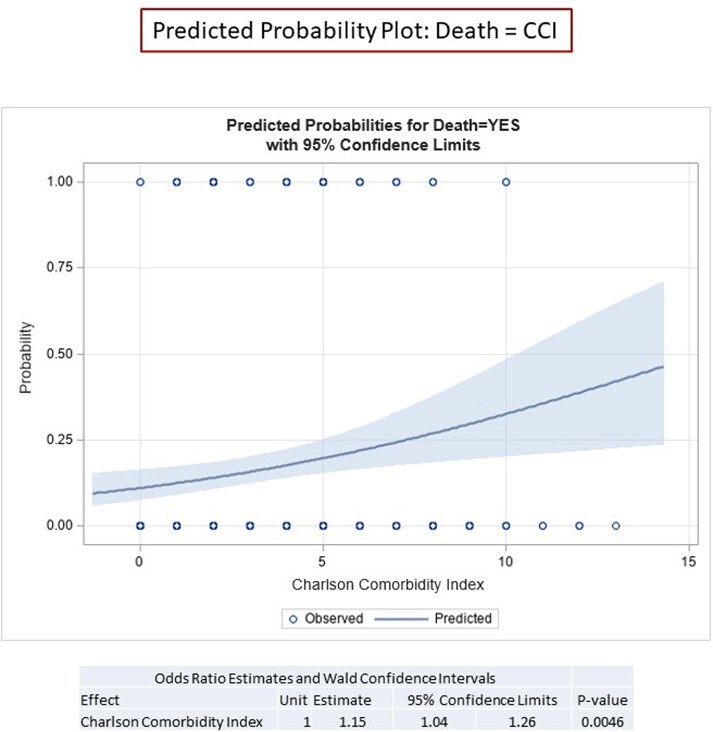

**Conclusion:**

This cohort study of hospitalized patients in an urban Veterans Affairs Medical Center found that race, ethnicity, and location of residence were not associated with COVID-19 disease severity and death, but median income and higher scores on the CCI predicted worse outcomes.

**Figure:**
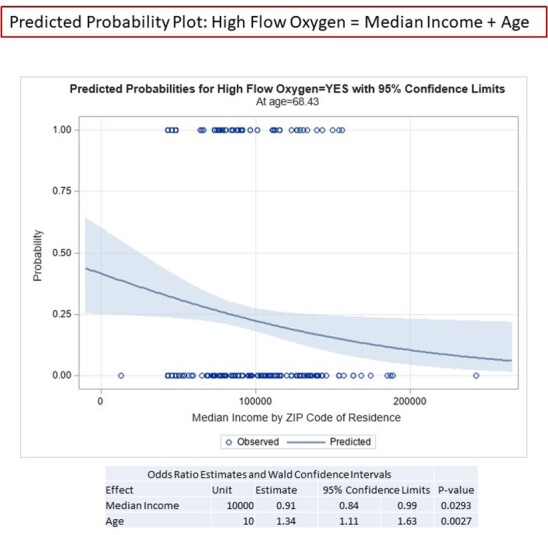

**Figure:**
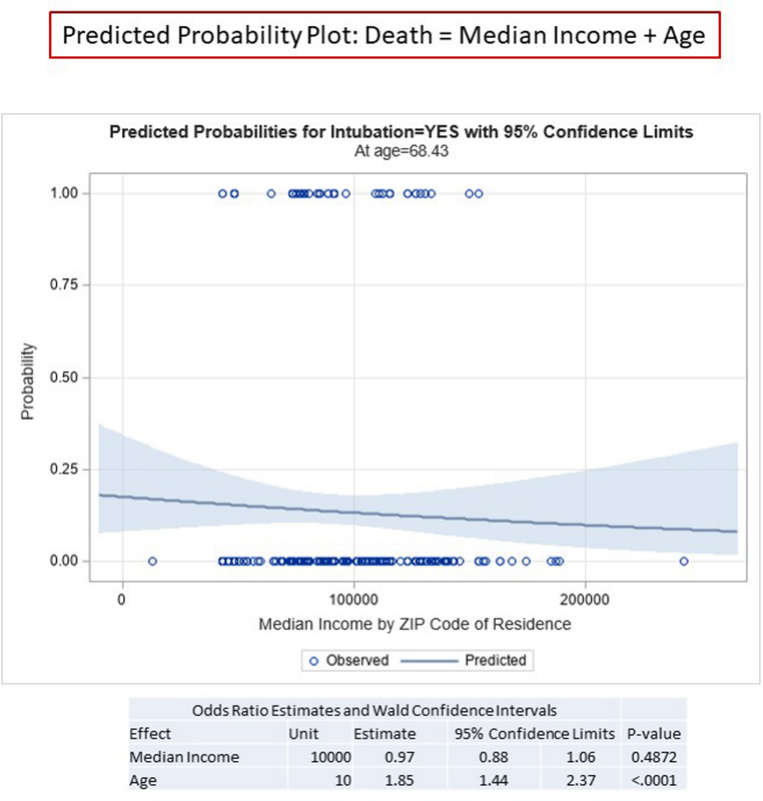

**Disclosures:**

**Pratish C. Patel, PharmD, BCIDP, AAHIVP**, VBI Vaccines: Stocks/Bonds

